# The Clinical Impact of Mean Vessel Size and Solidity in Breast Carcinoma Patients

**DOI:** 10.1371/journal.pone.0075954

**Published:** 2013-10-11

**Authors:** Lars Tore Gyland Mikalsen, Hari Prasad Dhakal, Øyvind S. Bruland, Bjørn Naume, Elin Borgen, Jahn M. Nesland, Dag Rune Olsen

**Affiliations:** 1 Department of Physics, University of Oslo, Oslo, Norway; 2 Department of Pathology, The Norwegian Radium Hospital, Oslo University Hospital HF, Oslo, Norway; 3 Department of Oncology, The Norwegian Radium Hospital, Oslo University Hospital, Oslo, Norway; 4 Institute for Clinical Medicine, University of Oslo, Oslo, Norway; 5 Faculty of Mathematics and Natural Sciences, University of Bergen, Bergen, Norway; Yokohama City University School of Medicine, Japan

## Abstract

Angiogenesis quantification, through vessel counting or area estimation in the most vascular part of the tumour, has been found to be of prognostic value across a range of carcinomas, breast cancer included. We have applied computer image analysis to quantify vascular properties pertaining to size, shape and spatial distributions in photographed fields of CD34 stained sections. Aided by a pilot (98 cases), seven parameters were selected and validated on a separate set from 293 breast cancer patients.

Two new prognostic markers were identified through continuous Cox regression with endpoints Breast Cancer Specific Survival and Distant Disease Free Survival: The average size of the vessels as measured by their perimeter (p = 0.003 and 0.004, respectively), and the average complexity of the vessel shapes measured by their solidity (p = 0.004 and 0.004). The Hazard ratios for the corresponding median-dichotomized markers were 2.28 (p = 0.005) and 1.89 (p = 0.016) for the mean perimeter and 1.80 (p = 0.041) and 1.55 (p = 0.095) for the shape complexity. The markers were associated with poor histologic type, high grade, necrosis, HR negativity, inflammation, and p53 expression (vessel size only).

Both markers were found to strongly influence the prognostic properties of vascular invasion (VI) and disseminated tumour cells in the bone marrow. The latter being prognostic only in cases with large vessels (p = 0.004 and 0.043) or low complexity (p = 0.018 and 0.024), but not in the small or complex vessel groups (p>0.47). VI was significant in all groups, but showed greater hazard ratios for small and low complexity vessels (6.54–11.2) versus large and high complexity vessels (2.64–3.06).

We find that not only the overall amount of produced vasculature in angiogenic hot-spots is of prognostic significance, but also the morphological appearance of the generated vessels, *i.e.* the size and shape of vessels in the studied hot spots.

## Introduction

Angiogenesis is a requirement for tumour growth beyond 1–2 mm^3^ [1]. The produced neo-vasculature usually exhibits a broad range of pathological features, including increased permeability, loss of architecture, and extensive inter tumour variability [2]–[4]. Once formed, it facilitates tumour growth and the metastatic process [1], [5].

Both angiogenesis [6], [7], disseminated tumour cells in the bone marrow (DTC) [8], and, in particular, the presence of tumour cells inside lymph- or blood vessels, i.e. vascular invasion (VI) [9], [10], are associated with an increased risk of future metastasis in breast cancer. Furthermore, angiogenesis has been found to be associated with DTC [11], [12], and influence the prognostic properties of both DTC and VI [13], [14].

Quantification of angiogenesis in the most vascular region of the tumour, i.e. the “hot-spot”, has been well studied and found to be a prognostic factor in a range of carcinomas [15]–[18] including breast cancer [6], [7], [13], [19]–[23]. The most common method is to manually count the number of vessels in a fixed size field (MVD) [19], [24]. However, due to its increased reliability, the Chalkley count (CC), a relative area estimate, has been recommended for use in solid tumours by an international consensus report on angiogenesis quantification [25]. Nevertheless, the methods have not provided the robustness and reproducibility required for clinical use [6], [25], [26]. In studies applying both MVD and CC to breast cancer, only the latter was a significant prognostic marker [16], [22], [27]. Thus, the specific vascular quantity is of clinical significance. However, morphometric characteristics of the microvessels or distribution parameters may have equal importance [28]–[32], but have so far not been elucidated in breast cancer [33], [34].

We have applied automatic image analysis to identify [35] and characterize vessels in photographed hot-spots from 394 patients with primary breast carcinoma. Based on results in a pilot data-set, seven vascular parameters were further evaluated for their prognostic impact and association with DTC and VI. We found that the markers representing the average size and shape complexity of the microvessels demonstrated prognostic significance.

## Materials and Methods

### Ethics Statement

Ethics approval for this study was obtained from the Regional Committee for Medical and Health Research Ethics (REC South East, Permit Number: S-97103). Written consent was obtained from all patients enrolled in the study.

### Patients and Tumours

CD34-immunostained sections from 394 patients out of the 920 enrolled in the Oslo Breast Cancer Micrometastasis Project from 1995 to 1998 were examined. The current study is based on a subset of the material previously reported on regarding the prognostic significance and clinico-pathological associations of DTC and tumour vascularity [13], [27], [36]–[38]. Cases were selected based on the availability and adequacy of primary tumour material for immunohistochemistry, with further exclusion of cases with disturbing CD34+ stromal elements (fibrocytes).

The material has a known significant impact from angiogenesis quantification using CC, but not MVD [27]. Clinico-pathological information was obtained from the database of the Oslo Breast Cancer Micrometastasis Study (Table 1). The patients were between 30 and 89 years (median 58). Seventy percent had received breast conservation surgery and 30% modified radical mastectomy. Out of 377 patients with information of non-surgical treatment, 22% received radiation therapy, 29% post operative adjuvant systemic therapy (chemotherapy and/or tamoxifen), while 25% received both. Patients undergoing preoperative chemotherapy or who developed metastasis prior to, or within one month of surgery were not included in the study. The follow-up time was from 1 to 125 months (median 85). Out of 385 patients with available information about relapse, 89 suffered systemic relapse; 36 patients (9%) had local recurrence. Out of the 391 patients, 69 died of breast cancer disease. Pathological tumour sizes were T1: 210, T2: 154 and T3–4: 18 cases. There were 237 node-negative patients. Tumours were graded according to Elston and Ellis [39]: G-I: 62, G-II: 143 and G-III: 88. Histologic types were according to WHO recommendations [9], there were 214 infiltrating ductal carcinoma not otherwise specified (IDC-NOS), 46 invasive lobular carcinoma (ILC) and 33 cases belonging to other types. VI was assessed in H&E-stained sections. The assessment of DTC by bone marrow aspiration from the iliac crest, its processing and evaluation has been previously described [37]. There were 90 cases with VI and 44 with DTC. The excluded cases (due to the presence of CD34+ fibrocytes [40], [41]) were associated with the ILC type, low tumour grade and VI-negativity, but were not significantly associated with survival end points (Table 1).

**Table 1:** Clinico-pathologic characteristics for patients in the different groups.

Dataset	Validation n = 293 (65.8%)			Pilot n = 98 (22.0%)				Excluded cases€ n = 54 (12.1%)			
Characteristics	n	Rel.Freq	group-%	n	Rel.Freq	group-%	p	n	Rel.Freq	group-%	p
Necrosis							0.840				0.098
Presence	27	(9.2%)	(77.1%)	8	(8.2%)	(22.9%)		1	(1.9%)	(2.9%)	
Absence	266	(90.8%)	(65.0%)	90	(91.8%)	(22.0%)		53	(98.1%)	(13.0%)	
Histologic types							0.088				<0.001
a: IDC NOS	214	(73.0%)	(67.1%)	80	(81.6%)	(25.1%)		25	(46.3%)	(7.8%)	
b: ILC	46	(15.7%)	(57.5%)	7	(7.1%)	(8.8%)		27	(50.0%)	(33.8%)	
c: Others*	33	(11.3%)	(71.7%)	11	(11.2%)	(23.9%)		2	(3.7%)	(4.3%)	
Histologic grade							0.805^£^				<0.001^£^
I	62	(21.2%)	(62.0%)	19	(19.4%)	(19.0%)		19	(35.2%)	(19.0%)	
II	143	(48.8%)	(63.6%)	49	(50.0%)	(21.8%)		33	(61.1%)	(14.7%)	
III	88	(30.0%)	(73.3%)	30	(30.6%)	(25.0%)		2	(3.7%)	(1.7%)	
ER							1.000				0.118
Positive	220	(75.1%)	(64.7%)	74	(75.5%)	(21.8%)		46	(85.2%)	(13.5%)	
Negative	73	(24.9%)	(69.5%)	24	(24.5%)	(22.9%)		8	(14.8%)	(7.6%)	
PgR							0.723				1.000
Positive	169	(57.7%)	(65.3%)	59	(60.2%)	(22.8%)		31	(57.4%)	(12.0%)	
Negative	124	(42.3%)	(66.7%)	39	(39.8%)	(21.0%)		23	(42.6%)	(12.4%)	
LN status							1.000				0.442
N0	177	(61.9%)	(64.8%)	60	(62.5%)	(22.0%)		36	(67.9%)	(13.2%)	
N+	109	(38.1%)	(67.3%)	36	(37.5%)	(22.2%)		17	(32.1%)	(10.5%)	
Inflammation							0.184				0.254
Minimal/mild	242	(82.6%)	(67.6%)	75	(76.5%)	(20.9%)		41	(75.9%)	(11.5%)	
Moderate/marked	51	(17.4%)	(58.6%)	23	(23.5%)	(26.4%)		13	(24.1%)	(14.9%)	
p53 expression							0.782				0.483
Positive	69	(23.5%)	(69.0%)	21	(21.4%)	(21.0%)		10	(18.5%)	(10.0%)	
Negative	224	(76.5%)	(65.1%)	76	(77.6%)	(22.1%)		44	(81.5%)	(12.8%)	
pT-status							0.844^£^				0.527^£^
T1	159	(54.3%)	(66.3%)	51	(53.7%)	(21.3%)		30	(60.0%)	(12.5%)	
T2	115	(39.2%)	(66.9%)	39	(41.1%)	(22.7%)		18	(36.0%)	(10.5%)	
T3	13	(4.5%)	(65.0%)	5	(5.3%)	(25.0%)		2	(4.0%)	(10.0%)	
T4	1	(0.3%)	(100%)	0	(0%)	(0%)		0	(0%)	(0.0%)	
DTC							1.000				0.177
Positive	33	(11.9%)	(61.1%)	11	(11.7%)	(20.4%)		10	(19.2%)	(18.5%)	
Negative	244	(88.1%)	(66.1%)	83	(88.3%)	(22.5%)		42	(80.8%)	(11.4%)	
Vascular invasion							0.267				0.012
Presence	72	(24.6%)	(75.8%)	18	(18.4%)	(18.9%)		5	(9.3%)	(5.3%)	
Absence	221	(75.4%)	(63.1%)	80	(81.6%)	(22.9%)		49	(90.7%)	(14.0%)	
HER-2 status							1.000				0.749
Positive	17	(5.8%)	(68.0%)	6	(6.1%)	(24.0%)		2	(3.7%)	(8.0%)	
Negative	274	(93.5%)	(65.2%)	92	(93.9%)	(22.4%)		52	(96.3%)	(12.4%)	
Systemic relapse							0.165				0.197
Yes	61	(21.3%)	(63.5%)	28	(28.6%)	(29.2%)		7	(13.2%)	(7.3%)	
No	226	(78.7%)	(66.1%)	70	(71.4%)	(20.5%)		46	(86.8%)	(13.5%)	
BC Death*							0.878				0.156
No	211	(72.0%)	(64.9%)	71	(72.4%)	(21.8%)		43	(79.6%)	(13.2%)	
Yes	51	(17.4%)	(68.9%)	18	(18.4%)	(24.3%)		5	(9.3%)	(6.8%)	
(Other/unknown)	31	(10.6%)	(67.4%)	9	(9.2%)	(19.6%)		6	(11.1%)	(13.0%)	
Age							0.983^τ^				0.652^τ^
Median		57.9			59.5				60.4		
Min		29.9			31.4				34.4		
Max		89.7			86.9				80.2		

€ Cases excluded due to background stains. p-values: Difference in group composition compared to the validation set; by Pearsons Chi-square exact test; Linear-by-linear association exact test (p-values marked with £); t-tests (p-values marked with τ).

* Other histologic types and unknown or unrelated causes of death were not included in the significance test.

### Immunohistochemistry

Paraffin-embedded blocks with representative tumour tissue were sectioned 4 µm thick, as previously described [13]. Briefly, sections were deparaffinised and microwaved in Tris/EDTA (pH 9.0), then treated with 0.03% hydrogen peroxidase for 5 min. The sections were incubated with a CD34 monoclonal murine antibody (IgG1) QBEND-10 (Monosan, the Netherlands) at room temperature for 30 min, then with a peroxidase-labelled polymer conjugated to goat anti-mouse antibody for 30 min, and finally with 3-3′-diaminobenzidine tetrahydrochloride for 10 min (Dako EnVision™+System Peroxidase (DAB) (K4007; DakoCytomation, CA, USA) and Dako Autostainer). Haematoxylin was used for counterstaining. Appropriate controls were included and showed satisfactory results.

### Image Analysis

**Figure 1:**
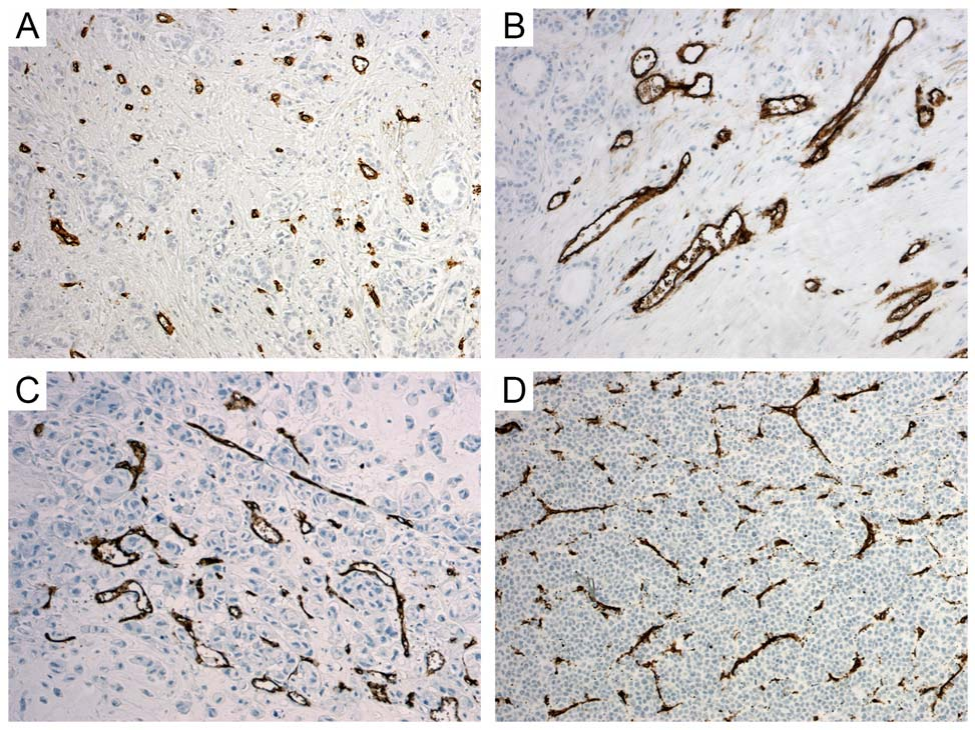
CD34 section case examples. A) High MVSμ; low MVPμ, MVA_Σ_ and MVD; B) Average MVSμ, high MVPμ and MVA_Σ,_ and low MVD; C) Low MVSμ, high MVPμ and MVA_Σ_, and low MVD; D) Low MVSμ, average MVPμ and MVA_Σ,_ and high MVD. High MVPμ values (large vessels) and low MVSμ values (high complexity shapes) contribute to poor prognosis; as well as high MVA_Σ_ values (high vascular area) [29], but MVD is inconsequential [29].

The image acquisition and field selection were performed as previously described [35]. Briefly, a low magnification scan in a light microscope was used to find the most vascular areas of the tumour. Three fields were photographed (image area 0.38 mm^2^; pixel pitch 0.34 µm). The image subjectively judged to be most vascular of the three was selected for each case. Cases with CD34-positive fibroblastic cells or spindle cells in the tumour stroma [40], [41] were excluded. Out of 445 photographed cases, 54 (12%) were excluded. Figure 1 shows examples of image quality and vascular characteristics.

**Figure 2:**
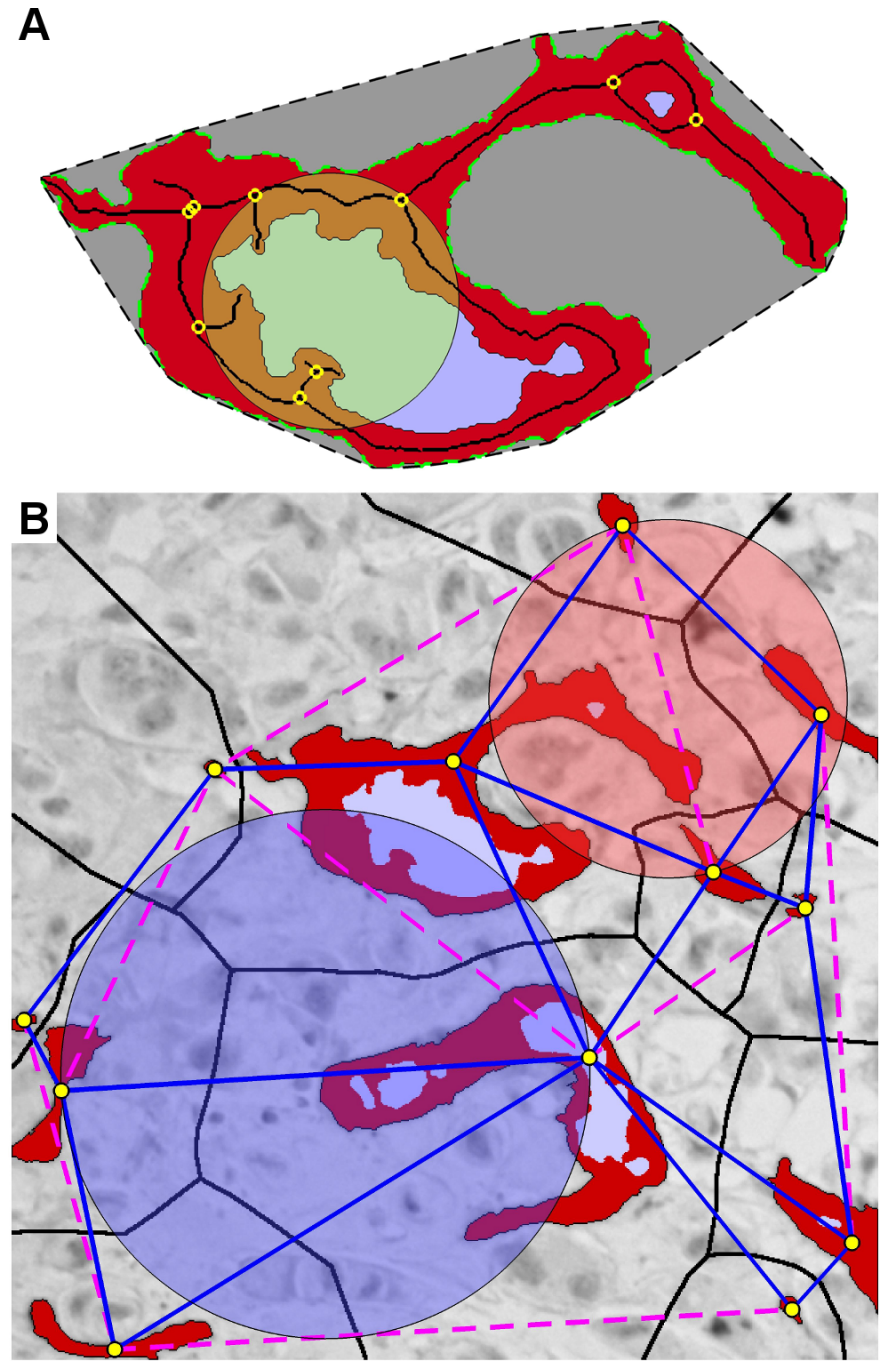
Illustration of the quantified vascular features. A) Individual vessel characterization: Endothelial area (red); luminal area (blue); vessel/vascular area (red + blue); vessel perimeter (stapled green); width (diameter of imposed yellow circle); skeleton (black line); branching points (yellow markers); convex hull (interior of stapled black line, i.e. vessel + gray). B) Contextual vasculature characterization: centre of vessel (yellow markers); Neighbouring vessels according to the Gabriel's Graph criterion (blue lines), i.e. all connections between vessels such that no other vessel centres are found within the circle spanned by the connecting line (see blue circle). Vertices which do not fit the criteria are not considered neighbours (magenta stapled lines; see red circle); equidistant watershed lines (black), each region within the black lines is closer to the enclosed vessel than any other vessel.

CD34 positive pixels were automatically identified in the images, according to previously reported specifications [35]. **Briefly,** stains were identified from dynamically calculated intensity thresholds that identified all dark pixels, and pre-set colour hue thresholds which rejected any pixel associated with haematoxylin (i.e. blue). While determining vessel profiles from the distributions of CD34^+^ pixels, only gaps in the stains wider than 1.0 µm were considered to be lumens, and only stained objects at least 3.5 µm wide, lumens included, were considered to be microvessels. Width was defined as the diameter of the largest inscribable circle, (Figure 2A). The remaining gaps and objects were considered to be noise, artefacts or similar, and were removed. The method achieved an intraclass correlation coefficient of r^2^ = 0.96 when the number of automatically identified vessels were compared to manual counting [35].

### Statistics

Survival analysis was performed using the endpoints Distant Disease Free Survival (DDFS) and Breast Cancer-Specific Survival (BCSS). Times were measured from date of surgery to event (systemic relapse and breast cancer death), or censured at last follow-up or unrelated death.

The data material was randomly split into two parts, pilot (25%) and validation (75%). The pilot cases (n = 98; events: 28 DDFS and 18 BCSS) were used to aid parameter selection, while validation cases (n = 296; events: 61 DDFS and 51 BCSS) were used to determine prognostic significance. Seven markers were validated (see parameter selection) using a common null hypothesis. The Bonferroni correction was applied with significance level α = 0.05 resulting in the test p≤β = α/7 = 0.0071. Marker validity was studied using Cox proportional hazard regression analysis (Cox regression) with statistically standardized continuous variables; hazard ratios (HR) correspond to an increase of one standard deviation. Markers that were significant (Wald test) for both DDFS and BCSS were considered prognostic. χ^2^-values are reported outside the context of hypothesis testing.

Prognostic parameters were further evaluated using median cut-off categorization. All further tests were done at the 0.05 level. Kaplan Meier analysis applied the logrank test; corresponding HRs were obtained using Cox regression for high-risk *vs* low-risk group. Associations with clinico-pathologic parameters were tested using the Pearsons chi-square or Linear-by-linear association exact test. Multivariate Cox regression was performed with parameters restricted to about 1 variable per 10 events.

Statistical calculations were carried out using IBM SPSS Statistics (version 19); IBM, Endicott, NY, US.

### Parameter Selection

Eighty-four pilot parameters were derived (*cf.* supporting information Pilot S1), and grouped together into six categories: a) vascular densities; b) vessel sizes; c) vessel shape characteristics; d) lumen measurements; e) distance parameters; and f) density scaling/fractal analysis parameters; (Figure 2). Using Cox regression χ^2^-values, each parameter category was sorted according to survival performance. Based on this and findings in the literature [30], [33], [42]–[45], six promising non-density parameters were selected for validation, ensuring no strong correlations among markers, or to vascular density. Additionally, MVSμ was added directly to validation, as there were no shape complexity markers in the pilot.

### Parameter names and definitions

The following markers were selected: MVPμ, the mean length of the vessels' outer perimeters; MVA_CV_, the vessel areas' coefficient of variation; MVA_rx4,_ the ratio of the total vascular area (MVA_Σ_) of the field to that of the highest scoring subfield corresponding to four times higher magnification; MV_luminal_, the fraction of vessel profiles with at least one discernible lumen. MV_scale_ is the average slope of the vascular content vs. field size curve in a double-logarithmic plot. The vessel profiles were first transformed to minimum connected strokes (skeleton) by removing their thickness (Figure 2A), reducing the association with MVA_Σ_. Square subfields were then randomly distributed across the image and the average vascular content (sum of stroke-lengths) of the fields computed using fifty different field sizes ranging from 3px to the image height. Low values are associated with uniform vessel distributions and small, simple vessels. MVSμ is the mean vessel solidity; i.e. the vascular area of the vessel divided by the area of its convex hull, i.e. the region with the shortest possible perimeter still containing the entire vessel (Figure 2A). High solidity is obtained for compact vessel profiles without branching, curvy shapes or otherwise concave regions, irrespective of elongation. ICD is the average intercapillary distance between neighbouring vessels, where neighbour is defined by the *Gabriels graph* criterion (Figure 2B). The calculation used vessel centre points, not the true distance between vessel boundaries.

## Results

### New vascular markers MVPμ, MVSμ

Seven parameters were tested in the validation material (Table 1). MVPμ and MVSμ demonstrated survival significance in continuous analysis for both endpoints (all p≤0.004). HRs were 1.3 and 1.4 for DDFS and BCSS respectively, for a one standard deviation increase in perimeter length, and 0.69 and 0.71 for an increase in solidity (i.e. reduced complexity) (Table 2). Trends were seen with MVA_CV_, MV_luminal_ and MV_scale_ for BCSS (p-values 0.026–0.081).

**Table 2:** Continuous Cox regression survival analysis of validation parameters.

		DDFS		BCSS	
		number of cases = 277		number of cases = 292	
		number of events = 60		number of events = 51	
*Parameter candidates from pilot:*		p	HR#	p	HR#
MVA_CV_	[size]	0.320	1.115	0.026	1.243
MVPμ	[size]	**0.003** *	1.286	**<0.001** *	1.369
MV_luminal_	[lumen]	0.198	1.178	0.060	1.287
MV_scale_	[scale]	0.102	1.235	0.081	1.278
MVA_rx4_	[scale]	0.540	1.139	0.633	0.935
ICD	[pattern]	0.760	1.038	0.904	1.016

*Additional parameter candidates:*					
MVSμ	[shape]	**0.004** *	0.706	**0.004** *	.685

* Significant at the Bonferroni corrected 0.05/7 level.

# Hazard ratios (HR) are for one standard deviation increase in the parameter value.

MVA_CV_: Coefficient of variation of the vessel areas. MVPμ: Mean vessel perimeter length; MV_luminal_: Fraction of vessels with open lumen. MV_scale_: Marker for the vascular density's dependency on the field size. ICD: Inter-capillary distance.

### MVPμ, MVSμ and Clinico-Pathological Parameters

MVPμ and MVSμ were found to be strongly associated with higher histologic grade, hormone receptor negativity, histologic type, presence of necrosis, moderate/marked inflammation, and p53 expression (Table 3). No associations were observed for tumour size, DTC, HER-2-status or VI.

**Table 3:** Clinico-pathological characteristics for patients and their relationship with the identified angiogenesis markers.

	MVPμ					MVSμ				
Characteristics	Low (n = 147)		High (n = 146)		p-value	Low (n = 147)		High (n = 146)		p-value
Necrosis					0.001					0.004
Presence	5	(18.5%)	22	(81.5%)		21	(77.8%)	6	(22.2%)	
Absence	142	(53.4%)	124	(46.6%)		126	(47.3%)	140	(52.6%)	
Histologic types					<0.001					0.010
a: IDC NOS	98	(45.8%)	116	(54.2%)		114	(53.3%)	100	(46.7%)	
b: ILC	34	(73.9%)	12	(26.1%)		15	(32.6%)	31	(67.4%)	
c: Others*	15	(45.5%)	18	(54.5%)		18	(54.5%)	15	(45.5%)	
Histologic grade					<0.001^£^					0.011^£^
I	40	(64.5%)	22	(35.5%)		24	(38.7%)	38	(61.3%)	
II	83	(58.0%)	60	(42.0%)		70	(49.0%)	73	(51.0%)	
III	24	(27.3%)	64	(72.7%)		53	(60.2%)	35	(39.8%)	
ER					<0.001					0.001
Positive	126	(57.3%)	94	(42.7%)		98	(44.5%)	122	(55.5%)	
Negative	21	(28.8%)	52	(71.2%)		49	(67.1%)	24	(32.9%)	
PgR					0.018					0.194
Positive	95	(56.2%)	74	(43.8%)		79	(46.7%)	90	(53.3%)	
Negative	52	(41.9%)	72	(58.1%)		68	(54.8%)	56	(45.2%)	
LN status					0.088					1.000
N0	96	(54.2%)	81	(45.8%)		88	(49.7%)	89	(50.3%)	
N+	47	(43.1%)	62	(56.9%)		55	(50.5%)	54	(49.5%)	
Inflammation					0.003					0.030
Minimal/mild	131	(54.1%)	111	(45.9%)		114	(47.1%)	128	(52.9%)	
Moderate/marked	16	(31.4%)	35	(68.6%)		33	(64.7%)	18	(35.3%)	
p53 expression					0.002					0.098
Positive	23	(33.3%)	46	(66.7%)		106	(47.3%)	118	(52.7%)	
Negative	124	(55.4%)	100	(44.6%)		41	(59.4%)	28	(40.6%)	
pT-status					0.282^£^					0.379^£^
T1	88	(55.3%)	71	(44.7%)		71	(44.7%)	88	(55.3%)	
T2	50	(43.5%)	65	(56.5%)		66	(57.4%)	49	(42.6%)	
T3	7	(53.8%)	6	(46.2%)		5	(38.5%)	8	(61.5%)	
T4	1	(100%)	0	(0%)		0	(0%)	1	(100%)	
DTC					0.581					0.138
Positive	15	(45.4%)	18	(54.5%)		21	(63.6%)	12	(36.4%)	
Negative	125	(51.2%)	119	(48.8%)		119	(48.8%)	125	(51.2%)	
Vascular invasion					0.280					0.135
Presence	32	(44.4%)	40	(55.6%)		42	(58.3%)	30	(41.7%)	
Absence	115	(52.0%)	106	(48.0%)		105	(47.5%)	116	(52.5%)	
HER-2 status					0.223					0.132
Positive	6	(35.3%)	11	(64.7%)		12	(70.6%)	5	(29.4%)	
Negative	140	(51.1%)	134	(48.9%)		135	(46.9%)	139	(50.7%)	
HoR status#					<0.001					0.002
Positive	134	(57.0%)	101	(43.0%)		107	(45.5%)	128	(54.5%)	
Negative	13	(22.4%)	45	(77.6%)		40	(69.0%)	18	(31.0%)	

# HoR– hormone receptor, combination of ER and PgR, positive if either is positive, negative if both are negative.

* Other histologic types were ignored for the purpose of determining significance.

p-value: Pearsons Chi-square exact test and Linear-by-linear association exact test (p-values marked with £). MVPμ: average perimeter length (low = small vessels). MVSμ: average vessel solidity (low = complex vessel shapes).

### MVPμ, MVSμ and Survival Analyses

**Figure 3:**
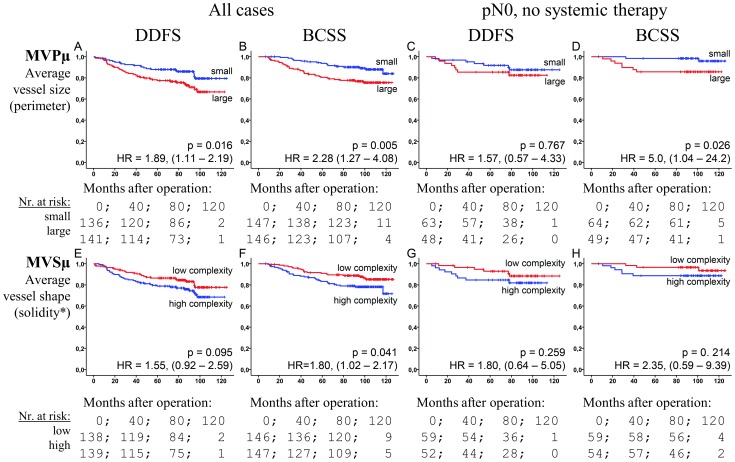
The prognostic associations of the dichotomized markers. The Kaplan-Meier plots of the median-categorized MVPμ (A–D) and MVSμ (E–H) data against DDFS (A, C, E, G) and BCSS (B, D, F, H) end-points in the set of all cases (A,B,E,F) and the set of node-negative no systemic therapy cases (C,D,G,H). p-values are by the log-rank test. Hazard ratios (HR) with 95-% confidence intervals in parentheses are for Cox regression of high-risk vs low-risk groups. *: Low and high solidity corresponds, respectively, to high and low vessel profile complexity.

While the loss of information incurred by grouping makes it optimal to use continuous data whenever possible [34], [46], grouping may be a necessary tool in clinical applications. Using median cut-offs and comparing the high risk group to the low risk, the HRs, for MVPμ and MVSμ respectively, were found to be 2.28 (p<0.005) and 1.80 (p = 0.041) for BCSS, and 1.89 (p = 0.016) and 1.55 (p = 0.095) for DDFS (Figure 3). In the node-negative no systemic treatment subgroup (n = 113), MVPμ showed significance for BCSS (HR = 5.0, p = 0.026) (Figure 3).

Multivariate survival analysis was performed separately for MVPμ and MSVμ. The models included clinico-pathological parameters that were significant in univariate survival analyses: histologic grade, LN-status, T-status, VI and hormone receptor status. In addition, systemic therapy was included. Neither marker came out significant in the multivariate model (Table 4).

**Table 4:** Multivariate Cox-regression.

	MVPμ				MVSμ			
	DDFS		BCSS		DDFS		BCSS	
	p	HR	p	HR	p	HR	p	HR
Histologic grade	0.083	1.565	0.343	1.311	0.080	1.564	0.260	1.380
LN status	0.002	2.901	0.002	3.331	0.002	2.902	0.001	3.553
pT status	0.002	1.901	0.000	2.442	0.002	1.901	0.000	2.385
Vascular invasion	0.002	2.351	0.002	2.634	0.003	2.349	0.005	2.451
HR	0.013	2.264	0.033	2.173	0.011	2.263	0.012	2.438
MVPμ	0.998	0.999	0.147	1.656				
MVSμ					0.989	1.004	0.472	1.257

MVP**μ** and MVS**μ** are dichotomized at the median-value; MVPμ is high *vs.* low; MVSμ is low *vs.* high. In addition to the shown parameters systemic therapy was included in the model. Neither MVPμ or MVSμ in their dichotomous versions were independently prognostic in the multivariate Cox model.

### MVPμ, MVSμ, DTC, VI and Survival

**Figure 4:**
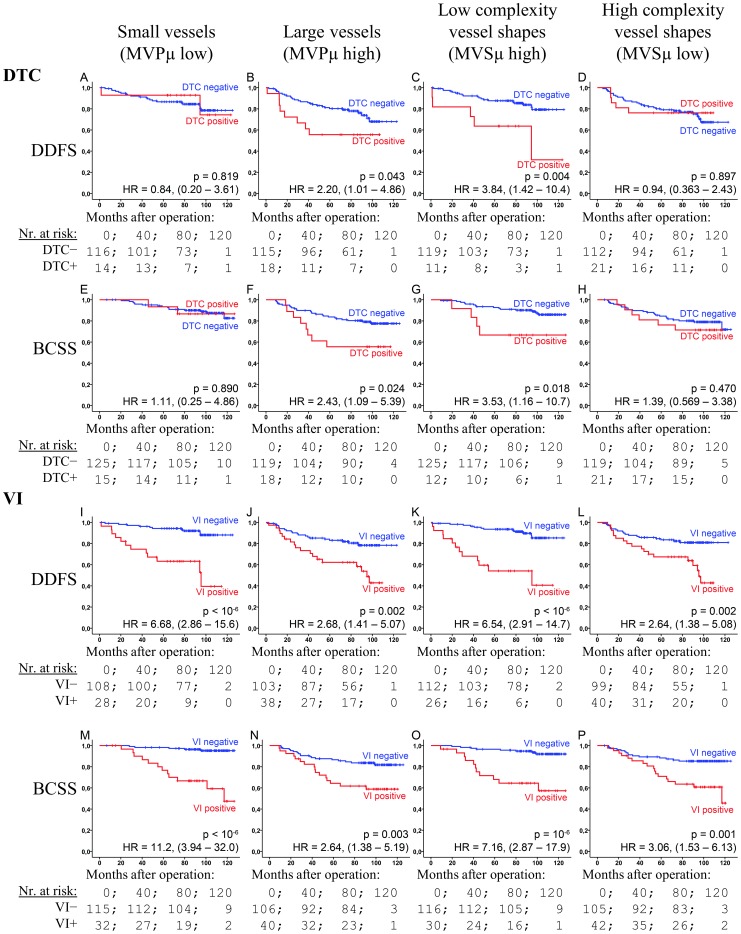
DTC and VI's prognostic dependency upon the vascular markers. The figure shows Kaplan-Meier plots of DTC (A–H) and VI (I–P) for the end-points DDFS (A–F and I–L) and BCSS (E–H and M–P) within vascular sub-groups (columns): Small vessels (A, E, I and M), large vessels (B, F, J and N), low complexity vessels (C, G, K, and O) and high complexity vessels (D, H, L and P). Gray curves: DTC or VI negative; black: Positive. HR: Cox regression hazard ratios with 95% confidence interval. p-values are by log-rank test.

Both MVPμ and MVSμ were found to strongly affect the prognostic properties of DTC and VI (Figure 4). In the large vessel group both DTC and VI had a moderately-high prognostic association (HRs = 2.21–2.68; p-values = 0.002–0.043). In contrast, the HR for VI was 6.68 (CI: 2.86–15.6) and 11.2 (3.94–32.0) in the small vessel group (for DDFS and BCSS, respectively), while DTC was insignificant (p>0.8). In the low vessel complexity group both DTC and VI demonstrated a stronger prognostic ability—all p<0.018; HR = 3.84 (1.42–10.4) and 3.53 (1.16–10.7) for DTC, and 6.54 (2.91–14.7) and 7.16 (2.87–17.9) for VI—than in the high vessel complexity group, in which VI demonstrated only moderately-high prognostic ability (HR = 2.64 and 2.06), and DTC none at all (p>0.47). HRs were in most cases (eleven out of sixteen) found to lie outside the confidence interval of the compared group (Figure 4).

### Relationship between Vascular Markers

MVPμ was the highest scoring of several strongly related vessel size measurements in the pilot. The others were the mean of the vascular area (MVAμ), endothelial area and vessel length (skeleton size). This result was repeated in the validation material (DDFS-χ^2^-values 6.3–7.7 vs. 9.1 for MVPμ; BCSS: 12.3–12.9 *vs.* 14.2; all p<0.012). Thus, all would be prognostic, but MVPμ performed somewhat stronger (trend). However, total perimeter length in the field (MVP_Σ_) was not associated to survival at all (χ^2^≤1.0); it had an eight times lower χ^2^-value than the total vascular area (MVA_Σ_).

The Pearson's correlation of MVSμ to MVPμ was found to be r^2^ = 0.35 (Kendall's τ^2^ = 0.21 for categorized data), while it was r^2^ = 0.16 to MVAμ. Despite being only moderately correlated, MVSμ and MVPμ were not independently significant in the bi-variate Cox regression model. MVSμ was, however, found to significantly add prognostic information to MVAμ for DDFS (p = 0.031) and a trend for BCSS (p = 0.071). Both MVPμ and MVSμ significantly added independent prognostic information to MVA_Σ_—a marker closely related to Chalkley counts—for both end-points (p-values: 0.013–0.028). MVA_Σ_ was positively correlated with MVPμ (r^2^ = 0.35) and negatively with MVSμ (r^2^ = 0.17).

## Discussion

In the present study, the mean vessel size and the mean vessel shape complexity, as measured by MVPμ and MVSμ respectively, were identified as strong prognosticators in invasive breast carcinoma patients. We observed a significantly reduced survival in patients with large vessels compared to those who had smaller vessels, especially in the continuous model (Table 2 and Figure 3). The finding is in accordance with previous reports for MVD and CC measurements in breast cancer [22], [27]. S Similarly, high vessel complexity was associated with poor prognosis (Table 2 and Figure 3). Furthermore, the microvessel size and vessel complexity had strong influences on the prognostic properties of DTC and VI (Figure 4). DTC has earlier been reported to have prognostic effect in high angiogenesis cases [13], [14], and VI in low angiogenesis cases [14]. Finally, the analysis of the relationship between the vascular markers shows that vessel size is a robust pathological marker with respect to different definitions, and that the prognostic ability of these markers is independent of vascular density. There is relevant work on similar markers in other cancers [29]–[31], [42], [47]–[54], but—to the best of our knowledge—no prognostic studies in breast cancer. There is, however, some literature on the association between vascular density measurements and DTC in breast cancer [11]–[14].

In this study, we used an automatic system to explore different vascular parameters that might be useful in evaluating tumour angiogenesis in a clinical setting. The size of the parameter-space far exceeded the statistical resolving power of the dataset; only a limited number of parameters could be analyzed. The literature concerning advanced quantifiers is limited and inconclusive [30], [34], [42], [55]. For this reason, we applied a pilot-validation setup, providing a basis for the selection of vascular descriptors, although at the expense of statistical power. Further, we applied strict significance criteria in order to provide a sound basis for inference.

The discovery of new independent vascular markers highlights a methodological challenge. The quantified fields are hot-spots selected in accordance with Weidner et.al. 's criteria [19], [24]. The fields were selected based on total vascular density. The question arise: Should angiogenesis markers be measured in the areas of the tumour where they are most extreme, i.e. MVPμ in the region with largest vessels, or are they prognostic because they characterize the hot-spot? This is relevant even with the traditional markers. In our case MVD was emphasised slightly higher than area when the hot-spot was selected, yet the area (CC) is by far the most prognostically powerful in breast cancer [16], [22], [27].

Hot-spot vascular density assessment is interpreted as a measurement of the biologically most active part of the tumour. Thus, to some extent, it reflects the tumours growth potential, as angiogenesis is a limiting factor for both proliferation and metastasis [5], [56], [57]. In contrast to MVD and CC, the new markers are at the individual vessel level. MVPμ and MVSμ relate to how functional the vessels are from a circulatory point of view. The mean vessel size has strong biological implications for the flow of blood through the vessels, and possibly the vessels ability to deal with mechanical stress, which is a driving force for acute hypoxia and confluent necrosis through arterial or venous infarct [56]. The MVSμ marker relates to several features including branching points, interconnectivity of vessels, and irregular vessel shapes in general; the marker has been used in anti-angiogenesis treatment studies to quantify vessel abnormality [58]. Disordered, heterogeneous and tortuous vascular networks are directly associated with hypoxia [59].

These characteristics are reflected, not only in a strong association with necrosis, but also in how the vascular morphology affects DTC's prognostic ability (Figure 4). DTC+ patients in the poorly functioning vascular groups (small or complex vessels) suffered no worse survival; DTC was only prognostic in the groups with large vessels, or low vessel complexity. Thus, vascular morphology can be used to screen patients for which DTC examination is beneficial. Possible explanations for DTC's lack of prognostic value in these groups are: Dormancy induced through increased biophysical stress (shear forces) on intravasated cells [60], increased time spent in the vasculature [61], or a tumour phenotype ill suited to initiate angiogenesis at the secondary site [62]. Finally, there is an association between vessel size and the size of tumour cell-aggregates entering the circulation; larger aggregates resulting in more metastases [63]. VI showed increased prognostic ability in groups associated with low angiogenesis, in accordance with earlier findings [14]. In breast cancer, VI has been found to primarily be of the lymphovascular type [64].

MVPμ and MVSμ were not found to be associated with physical tumour extension characteristics; T-status, LN-status, VI, and DTC status were insignificant. Strong associations were, however, observed for tumour grade, ER, necrosis, inflammation, and histologic type (Table 3). Thus, the markers' associations seem linked to the biology of the tumour, than physical extent. These associations differ from those reported for CC, which is strongly associated with T-status and VI [13], [21], [65].

In conclusion, we have identified two new prognostic vascular markers, MVPμ and MVSμ, pertaining to features at the individual vessel level. Not only is the overall amount of neo-vascularisation of prognostic value in breast cancer, but also morphological characteristics of the produced vessels. These new parameters add into our further understanding the role of angiogenesis in tumour progression, dissemination and metastasis. We find that the measurement of average vessel size and vessel shape in the hot-spot provides new prognostic information.

## Supporting Information

Pilot S1 **Description of pilot parameters.**
(DOC)
