# Supplementary material for: The Clinical Impact of Mean Vessel Size and Solidity in Breast Carcinoma Patients
Source: PLoS One. 2013 Oct 11;8(10):e75954. doi: 10.1371/journal.pone.0075954 (PMC3795733; doi:10.1371/journal.pone.0075954)
Supplement: Pilot S1 — Description of pilot parameters. (DOC) [file pone.0075954.s001.doc]

# Supplementary material

## Pilot parameters description

In total 84 parameters were defined and calculated in the pilot data set. The parameters were related to six different types of features: a) vascular density (8 markers); b) vessel sizes (13); c) vessel shape characteristics (5); d) lumen measurements (9); e) distance parameters (30); and f) density scaling/fractal analysis parameters (19). Multiple markers were strongly related, e.g. endothelial and vascular area; vessel area and vessel length; or Distance between vessels and area closest to each vessel, the effective number is thus less. The eight vascular density markers were derived and correlated with other pilot markers, but not considered for validation.

### a) Total vascular density (8) and b) vessel size measurements (13)

Vascular density and vessel size measurements were extracted from parameter histograms. In addition to the number of vessels (MVD), four feature histograms were analysed: Endothelial area (lumens excluded), vascular area (lumens included), vessel perimeter lengths, and simulated Chalkley counts. Automated Chalkley counts were calculated by convolving an image of the Chalkley count graticule (scaled to desired magnification, graticule points reduced to 1 px) with the vessel map for 50 different rotations of the graticule; convolution was performed using the Fourier theorem.

Derived parameters: MVD (num of objects); Chalkley count (eq. magnification: x200 and x400); endothelial area histogram (sum, mean, max, CV); vascular area histogram (sum, mean, max, CV); vascular perimeter (sum, mean, max, CV); vessel length (sum, mean, max); and vessel thickness (sum, mean, max);

### c) vessel shape characteristics (5)

Four histograms were investigated: stain eccentricity, vessel length, vessel width and the number of branching points. The stain eccentricity is the eccentricity of the ellipse which statistically is most similar to the object (same second order statistical moments). Length was defined as the long side of the skeletons bounding box, branching points as skeletal branching points, and thickness was the area divided by the length (Figure 2).

Derived parameters: Statistical eccentricity histogram (mean, CV); branching points (sum, mean, max).

### d) Lumen measurements (9)

The following lumen distributions were investigated: Area of the lumens (mean, max, CV); Ratio of luminal to endothelial area (mean, max), and lumen perimeter (mean). In addition the following parameters were derived: Number of lumens, number of vessels with lumen and fraction of vessels with lumen.

### e) Vessel map distance parameters (30)

The distance from each pixel in the image to the nearest vessel was identified, as was the area closest to each vessel. From the distances from tumour regions to nearest vessels a cumulative histogram was calculated. This showed how large fraction of the image was further than x µm away from the vessel, and the following features extracted: The 50^th^ percentile, 79^th^ percentile, and 90^th^ percentile values, as well as the slope at the 50^th^ percentile, the distance increase needed to get from the 90^th^ to the 99.5^th^ percentile. In addition the distance transform was used to derive the fraction of the area that was further than 70µm away from the nearest vessel. From the regions closest to each vessel, histograms parameters of the area (max, CV, skewness), eccentricity (mean, CV), and solidity (mean, CV) were extracted. In addition the ratio of the 67^th^ percentile to the 33^rd^ percentile of the sorted cumulative region area histogram was calculated, as well the corresponding ratio for the number of vessels needed to reach the respective percentiles. Furthermore, applying graph theory, Gabriel’s graph (all connections between vessels such that no other vessel centres are found within the circle spanned by the connecting line (Figure 2B)) and the Euclidean minimum spanning tree (the shortest possible graph containing all vessels) were calculated (Figure 2B). The following features were extracted: number of branches (i.e. connections), number of branches per vessel (Gabriel’s graph only), sum of branch lengths, average branch lengths per vessel, and the histogram parameters sum, mean, CV, skewness and kurtosis.

### f) Dependency on field size (19)

This category contains one marker which is manually obtainable: i.e. the vascular density at full field divided by the density of the highest coring subfield equivalent to four times higher magnification. This was performed for microvessel density, endothelial area, and vascular area. For the automated Chalkley counts the ratio of x200 to x400 was used.

In addition 12 more markers were obtained from fractal analysis or adaptations of this, i.e. from curve fitting (slope, intersect, goodness of fit) of double logarithmic plots of the vascular content versus the field size. The basic method (sandbox algorithm ) calculates the average vascular content around each vessel looking at progressively larger regions around each vessel. This was applied to images of the vessels’ mass centres, providing the fractal dimension of the vessel count, and to the vessels’ skeletons with curve fits of the low field sizes only providing the fractal dimension of the vessels skeletons and branching. In additions two related measurements were conducted similar to that above, but instead of sampling the vascular content around vessels, it measures the average vascular density throughout the image. This was performed using the endothelial and vascular skeletons (trimming away side-branches shorter than 7µm) and fitting across all field sizes, and using vessel mass centres fitting only the medium to large field sizes.

## References

1. Gazit Y (1997) Fractal characteristics of tumor vascular architecture during tumor growth and regression. Microcirculation 4: 395.
